# Photoperiodic responses of Sahelian malaria mosquitoes *Anopheles coluzzii* and *An. arabiensis*

**DOI:** 10.1186/s13071-017-2556-z

**Published:** 2017-12-27

**Authors:** Diana L. Huestis, Monica L. Artis, Peter A. Armbruster, Tovi Lehmann

**Affiliations:** 1 Laboratory of Malaria and Vector Research, National Institute of Allergy and Infectious Diseases, National Institutes of Health, Rockville, MD USA; 2 Department of Biology, Georgetown University, Washington, DC USA

**Keywords:** *Anopheles coluzzii*, *Anopheles gambiae* (*s.l.*), *Anopheles arabiensis*, African malaria mosquito, Dormancy, Diapause, Aestivation, Survival, Longevity, Body size, Dry season, Life history, Seasonality

## Abstract

**Background:**

Throughout large parts of sub-Saharan Africa, seasonal malaria transmission follows mosquito density, approaching zero during the dry season and peaking during the wet season. The mechanisms by which malaria mosquitoes survive the long dry season, when no larval sites are available remain largely unknown, despite being long recognized as a critical target for vector control. Previous work in the West African Sahel has led to the hypothesis that *Anopheles coluzzii* (formerly M-form *Anopheles gambiae*) undergoes aestivation (dry-season diapause), while *Anopheles gambiae* (*s.s.*) (formerly S-form *An. gambiae*) and *Anopheles arabiensis* repopulate each wet season via long-distance migration. The environmental cues used by these species to signal the oncoming dry season have not been determined; however, studies, mostly addressing mosquitoes from temperate zones, have highlighted photoperiod and temperature as the most common token stimuli for diapause initiation. We subjected newly established colonies of *An. coluzzii* and *An. arabiensis* from the Sahel to changes in photoperiod to assess and compare their responses in terms of longevity and other relevant phenotypes.

**Results:**

Our results showed that short photoperiod alone and to a lesser extent, lower nightly temperature (representing the early dry season), significantly increased longevity of *An. coluzzii* (by ~30%, *P* < 0.001) but not of *An. arabiensis*. Further, dry season conditions increased body size but not relative lipid content of *An. coluzzii*, whereas body size of *An. arabiensis* decreased under these conditions.

**Conclusions:**

These species-specific responses underscore the capacity of tropical anophelines to detect mild changes (~1 h) in photoperiod and thus support the role of photoperiod as a token stimulus for *An. coluzzii* in induction of aestivation, although, these responses fall short of a complete recapitulation of aestivation under laboratory conditions.

**Electronic supplementary material:**

The online version of this article (10.1186/s13071-017-2556-z) contains supplementary material, which is available to authorized users.

## Background

Although many strides have been made in recent years, malaria remains a global challenge, with hundreds of millions of cases, near 450,000 deaths, and billions of dollars of lost productivity each year [1] mostly in sub-Saharan Africa. Mosquitoes in the genus *Anopheles* are the primary vectors of human malaria, yet much about their basic ecology still remains unknown. *Anopheles gambiae, An. coluzzii* and *An. arabiensis* are widespread across the African continent, inhabiting a wide range of habitats with environmental conditions that vary both spatially and temporally [2–5]. Specifically, rainfall amount and distribution is highly variable across their range, with large portions having a 4–7 months-long dry season [6–10]. Because anopheline mosquitoes have a relatively short lifespan, estimated at 2–5 weeks; [11–15] and the aquatic stages tolerate desiccation only for a few days [16, 17], the mechanism(s) underlying their persistence without surface water in extensive regions with dry spells exceeding 4 months is one of the longest unanswered questions in medical entomology [6, 18–22]. Two competing hypotheses have been proposed to explain the persistence of Anophelines in regions with extended dry seasons: (i) long-distance migration (LDM) from areas with permanent larval sites to seasonally arid areas after the rainy season begins or (ii) extension of life in local populations via aestivation, (summer diapause) while hidden in local shelters [6, 19–23]. Knowledge of the mechanism(s) of persistence used by each vector species could yield effective intervention strategies for reducing malaria transmission across vast areas with seasonal cycles.

In the Sahel (latitudes 12–18°), it is hypothesized that *An. coluzzii* (previously M-form *An. gambiae*; [24]) survive the dry season by aestivating locally, while *An. gambiae* (previously the S-form) and *An. arabiensis* persist via annual long-distance migration [8, 20, 25–28]. The physiological mechanisms of mosquito aestivation and its initiation and maintenance in the field have been studied in recent years, yet are still largely unknown [21, 29–34]. However, if aestivation is similar to other cases of insect diapause, it may be initiated by predictable, seasonal shifts in photoperiod, temperature, or other environmental conditions [35–45]. In the Sahel, rainfall sustaining surface waters for mosquito breeding only occurs between June and October. The maximal photoperiod change between seasons is ~2 h and during the supposed transition period (late October), inferred based on the species-specific disappearance of *An. coluzzii* [8], the photophase is only 15–20 min shorter than the scotophase (11.75:12.15 h, L:D, respectively). During that time, the daily rate of decrease in photoperiod is the highest (45 s/day) and both RH and nightly temperatures start falling [21, 33]. Seasonal shifts in photoperiod are typically associated with temperate climates, yet insects and other organisms are sensitive to even subtle shifts in photoperiod which occur in the tropics [45, 46]. For example, at latitude 9°, the beetle *Stenotarsus rotundus* responds to photoperiod by degeneration or development of flight muscles [47], the African stonechat at the equator probably uses subtle differences in the time of sunrise and sunset [48], and the spotted antbird detects changes in photoperiod as slight as 20 min and responds in dramatic changes in reproduction [49]. Additionally, a combination of shorter day length and lower temperatures, typical of the dry season were key to induction of aestivation in the Sahelian bruchid beetles, in which the adults seek (unknown) shelters during the long dry season [50, 51]. Thus, it is reasonable that photoperiod, including the daily change in photoperiod, possibly coupled with temperature change, could be the seasonal cue(s) used by mosquitoes to initiate aestivation in the Sahel. Uncovering the factors which stimulate mosquitoes to begin, maintain, and terminate aestivation could lead to better prediction of disease-transmission seasons and may also provide novel opportunities for vector control [21, 34, 52].

To test if *An. coluzzii* mosquitoes use photoperiod and temperature as cues to initiate and/or maintain aestivation, we measured changes in their longevity and other relevant life-history traits under dry-season as compared to wet-season photoperiod and temperature conditions. The present study extends previous results showing that under short photoperiod, *An. gambiae* (G3 colony) had larger body size and larger amount of cuticular hydrocarbons standardized for body size [34]. Here, we included the presumably non-aestivating West-African *An. arabiensis* as a control comparison based on the rationale that *An. arabiensis* would not increase its longevity in response to the change in photoperiod. The strategy used by of *An. arabiensis* to persist through the dry season is less clear; however, evidence from Mali suggests that persistence in this population relies on long-distance migration during the rainy season and therefore it is not expected to respond to such changes or its response would not agree with that of *An. coluzzii* [6–8, 19–21, 28, 32]. In addition to longevity, we predicted that other aestivation-relevant phenotypes, such as body-size and nutritional reserves would be affected by changes in photoperiod in *An. coluzzii* but not in *An. arabiensis* (Table 1). These hypotheses regarding the expected change in response to photoperiod and temperature range in strength from the strongest which is for adult longevity [20], through the moderately-strong predicted change in body size [29, 30, 34] based on the larger body size of *An. coluzzii* mosquitoes in the dry season, to weaker predictions about body mass and developmental time, which have not been assessed previously. A larger body size is expected to confer higher tolerance to desiccation due to smaller surface/volume [29]. To test these hypotheses, new laboratory colonies of these species were established from a Sahelian field site. Photoperiod conditions were selected to mirror those which naturally occur at that source location during the wet season (June-July), the transition period between the wet and the dry season (October-November), and during the dry season (January-February). Our results revealed that *An. coluzzii* responded to the changes in photoperiod more than its sibling species *An. arabiensis*. *An. coluzzii* expressed changes in development time, body size (and body mass) at emergence, as well as in longevity. The responses agree with predictions based on evidence of aestivation in the dry season, but they were modest in size, suggesting that additional conditions may be critical for full expression of aestivation.

**Table 1:** Predicted and observed responses of females *Anopheles coluzzii* and *An. arabiensis* to simulated dry season as opposed to wet season conditions in Experiment 1 (photoperiod alone) and Experiment 2 (photoperiod and temperature)

Trait	Predicted: dry vs wet conditions		Observed: dry vs wet conditions			
	*An. coluzzii*	*An. arabiensis*	*An. coluzzii*		*An. arabiensis*	
			Experiment 1	Experiment 2	Experiment 1	Experiment 2
Longevity (Adult)	**Change: increase**	No change	Increased √	Increased √	Not changed √	Not changed^*^ √
Body Size (WL)	**Change: increase**	No change	Increased √	Increased^*^ √	Decreased √	Decreased √
Body mass (dry)	Change: increase	No change	Increased √	Increased^*^ √	Decreased √	Decreased √
Lipid content	Change: increase	No change	Not changed	Increased √	Not changed √	Not changed √
Larval development Time	Change: increase	No change	Decreased	Not changed	Increased	Not changed √

*Notes*: Prediction is bold reflect strongest predictions based on previous studies in the field. Checkmarks identify results of the experiment that are consistent with aestivation in *An. coluzzii* but not in *An. arabiensis*. Responses that have partly matched predictions were marked with a ‘^*^’

## Methods

### Mosquito colonies

New laboratory colonies of *An. coluzzii* and *An. arabiensis* were established in November 2012 from blood-fed and gravid females collected in the village of Thierola, Mali, where previous studies on the dry-season ecology of these mosquitoes have been conducted since 2008 [8, 20]. Wild females that laid eggs and two of their offspring were identified to species using the PCR followed by *Hha*I digestion as previously described [53]. Species identity were checked and confirmed at generations 1, 2 and 4. Mosquitoes of each species were maintained for 4 generations prior to the start of the first experiment to increase abundance and reduce environmental and maternal effects from the field. Mosquitoes were kept under 12:12 L:D cycle, 27 °C, and 75% RH. Larvae were maintained in pans containing ~2.5 cm of dechlorinated water and fed daily grounded fish food TetraMin Baby fish food (Spectrum Brands, Inc., Madison, WI, USA). Adults were provided with 10% Karo syrup that was refreshed daily. When the mosquitoes were 5 days old, they were offered blood meal on a human arm (generation 1 and 2) and thereafter on mice. Mosquitoes were allowed to feed for 15 min and were given an additional 5 min if the feeding rate was < 50%.

### Experimental design

In the first experiment, hereafter referred to as “photoperiod alone” or “Exp. 1”, we used custom-built photoperiod chambers described previously [42] to simulate the daily photoperiod and daily changes in photoperiod which occur in our field area. Each photoperiod chamber was equipped with full-spectrum fluorescent bulbs and controlled by ChronTrol XT-4 timers (ChronTrol Corp., San Diego, CA, USA). We simulated 3 light regimes characteristic of different seasonal periods. First, the early wet season photoperiod (July through August), was simulated with an initial 13:11 (L:D) that decreased by 10 s/d. Second, the photoperiod during the transition between the wet and the dry seasons (October through November, when *An. coluzzii* numbers fall rapidly, as presumably they move to shelters for aestivation; [8, 25]) was simulated with an initial 12:12 (L:D) that decreased by 45 s/d for 40 days, after which the rate of decrease was decreased by 2 s/d every 2 days until the rate was 10 s/d. Finally, the early dry season photoperiod (January through February) was simulated with an initial 11:13 (L:D) that increased by 10 s/d. Photoperiod treatments were designed based on calculated day-length (and its daily shift) for the latitude and longitude of Thierola, Mali (13.659°N, 7.215°W), using a freely available sunrise- and sunset-calculating resource which measures day length to the second (https://arachnoid.com/lutusp/sunrise/).

After the colonies had been expanded for 4 generations (above), cages of 6–9 day-old adult mosquitoes (~400 mosquitoes/cage; 2 cages/species) were fed for 30 min on an anesthetized Swiss-Webster mouse, with feeding repeated 2 days later to generate large egg batches. Egg dishes (moistened filter paper shaped into a funnel and placed in a paper cup) were placed into the cages 3 days after the second feeding and females were allowed to oviposit overnight. These eggs were counted and randomly divided into 6 batches of 400–600 eggs per species. These 6 batches were randomly assigned to one of three photoperiod treatments: wet season, wet-dry transition, or dry season; each treatment therefore had two replicates per species. Egg batches were flooded with water in small plastic pans within the photoperiod chambers and larvae were reared to pupation. Pupae were removed daily into a new container and newly emerged adults collected and counted each day before being placed into a larger, replicate-specific adult cage. Pupal collection continued for each replicate until at least 95% had emerged. Adults were provided continuous access to cotton balls soaked in 10% Karo syrup, which were replaced daily, following observations that some plants (e.g. *Acacia* spp. and *Azadirachta indica*) flower during the dry season. Larval pans and adult cages were randomly rearranged daily within each photoperiod chamber to minimize positional effects.

To assess if there were any differences in body size or lipid content between the treatments, 6 newly emerged females were collected on the 4th day of emergence for each group (the peak of the emergence curve) and preserved via immediate desiccation over silica gel. For each individual, wings were mounted and wing-length measured as previously described [54], desiccated carcasses weighed to the nearest 0.001 mg using a Cahn C-31 electrobalance (Cahn Instruments, Cerritos, CA, USA), and total lipid content was quantified using the vanillin-phosphoric acid method of Van Handel [55].

Mosquitoes were provided with an anesthetized Swiss-Webster mouse for blood-feeding on the 8th day after the peak emergence date and on day 10. Water for oviposition (see above) was provided for all cages 4 days after the second feeding. After the first gonotrophic cycle, all cages were provided identical access to blood-feeding (once per week for 10 min), but only the wet-season photoperiod treatment groups were given access to oviposition water, to better simulate conditions found during the dry-season and the transition from wet season to dry season [20, 25, 32]. To assess mortality, dead mosquitoes from each cage were counted and preserved daily until the cage was empty.

The second experiment, hereafter referred to as “photoperiod and temperature” or “Exp. 2”, was conducted as a follow up to evaluate the effect of temperature as an additional cue for the dry season life-history changes [40]. This experiment was based on the well-established rationale that photoperiodic cues can be affected by temperature [45, 56]. The design of this experiment was similar to the photoperiod alone experiment, but we only used the wet-season and dry-season photoperiods, i.e. the transition treatment was dropped, based on the results that experiment (see below), which showed little effect of the wet-to-dry season transition treatment. Photoperiod effects are often evaluated under “unambiguous photoperiod conditions”, referring to more extreme regimes than typically experienced by natural populations (see [41] and references therein). To better represent the natural conditions [21, 33], showing lower temperatures and greater daily variation of temperatures in the early to mid-dry season, we included wet-season (27 °C) and dry-season (25 °C nighttime and 29 °C daytime with 1.5 h of transition on each side) temperature treatments, which were crossed with the two remaining photoperiod treatments (wet-season and dry-season) in all combinations. The average daily temperature was equal (27 °C) across the treatments of the dry and wet seasons and RH was kept at 75% throughout both experiments. Artificial plastic “shelters” were also placed in one corner of the cage (Additional file 1: Figure S1) for all replicates to provide an opportunity for mosquitoes to seek shelter, as has been proposed to occur during the dry season [25].

### Statistical methods

A measure of mean egg-to-adult developmental time was calculated for each cage as the weighted daily average. The calculated mean emergence date for each cage was then used as the starting date for calculating adult longevity. Lipid content was calculated as the ratio of total lipid (μg) to the mosquito dry weight (μg). Mortality during the first day following introduction of mosquitoes to the cages was assumed to be a result of handling (accidental death). Survival analysis was performed using Proc Lifetest and Proc Phreg [57], testing relationships between longevity and treatment factors using univariate survival analysis in Proc Lifetest (comparing Kaplan-Meier survival functions between levels of each factor and employing Wilcoxon tests. Multivariate analysis using the Cox proportional hazard regression (Proc Phreg [57]) was carried out with stratification by experiment. Analysis of variance (ANOVA) was conducted using Proc GLM in SAS to test the effect of treatment on development time, body size, and whole-body lipid content. Significant differences between treatments were evaluated using the REGWQ test, unless the interaction(s) were significant; in which case least square means were used.

## Results

### Development time (egg deposition to adult ecolosion)

A total of 3481 (1733 females) and 4460 (2245 females) adult mosquitoes emerged in the photoperiod-alone experiment (Exp. 1) and the photoperiod and temperature experiment (Exp. 2), respectively. In both experiments, males developed faster than females (Exp. 1: *F*_(1,3474)_ = 36.6, Exp. 2: *F*_(1,4450)_ = 30.8, *P* < 0.01) in analyses within species across treatments. In both experiments, differences between species, treatments, and species-by-treatment interaction were all significant (Exp. 1: *F* > 31, Exp. 2: *F* > 4.5, *P* < 0.037).

#### Photoperiod only: Experiment 1

**Fig. 1:**
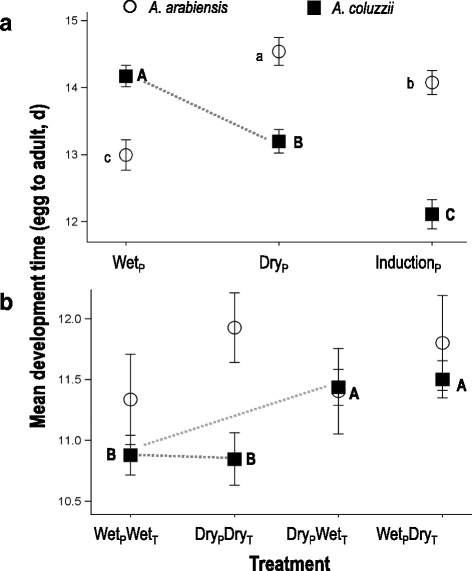
Mean developmental time for female *A. arabiensis* (white circles) and *A. coluzzii* (black squares) under (**a**) three photoperiod treatments (Experiment 1): wet season, dry season, and wet to dry transition (induction), and (**b**) four photoperiod-temperature treatment combinations (Experiment 2). The subscript ‘P’ and ‘T’, denote photoperiod and temperature conditions, respectively. Mean ± 2SE are given and significantly different values within each species are designated with letters (REGWQ groupings). The grey dotted line denotes the critical comparison between wet-season and dry-season *A. coluzzii* in each experiment

Development time of female *An. arabiensis* increased by 1.5 days, from 13.0 d to 14.5 d, with decreasing day-length from the wet to the dry season (*F*_(1,775)_ = 91.4, *P* < 0.001; Fig. 1a). In contrast, female *An. coluzzii* developing under wet-season conditions had the longest development time (14.2 d) while those developing under the induction (wet-to-dry transition) photoperiod had the shortest (12.1 d, *F*_(__1,952)_ = 66.9, *P* < 0.001; Fig. 1a), amounting to 15% reduction in developmental time.

#### Photoperiod and temperature: Experiment 2

Development time of female *An. arabiensis* differed minimally across all 4 photoperiod-temperature combinations (*F*_(__3,415)_ = 3.1, *P* = 0.026; Fig. 1b) whereas, for female *An. coluzzii*, the effect of treatment was highly significant (*F*_(__3,1822)_ = 16.4, *P* < 0.0001). However, females developing in the photoperiod-temperature “matched” treatments [i.e. wet-season photoperiod with wet-season temperature (10.9 d), and dry-season temperature with dry-season photoperiod (10.8 d)] were not significantly different from each other (*F*_(__1,1822)_ = 0.5, *P* = 0.82). Moreover, the development time of the two “matched” treatments were significantly shorter (*F*_(__1,1822)_ = 47.3, *P* < 0.0001) than the two “mismatched” treatments (wet-season photoperiod with dry-season temperature and dry-season photoperiod with wet-season temperature), which were not different from each other (*F*_(__1,1822)_ = 0.4, *P* > 0.55; Fig. 1b).

### Body size and lipid reserves

At adult emergence, positive correlations were found between female body size (measured as wing length), dry mass, and lipid mass across species and treatments (*An. arabiensis* Exp. 1: 0.34 < *r* < 0.69, 0.002 < *P* < 0.15, *n* = 20–23; Exp. 2: 0.81 < *r* < 0.85, *P* < 0.0001, *n* = 20; and *An. coluzzii* Exp. 1: 0.54 < *r* < 0.95, *P* < 0.019, *n* = 18–20; and Exp. 2: 0.56 < *r* < 0.64, *P* < 0.0001, *n* = 38). However, the relative lipid content (proportion of lipids from total dry mass) was negatively correlated with body size in *An. arabiensis* (Exp. 1: -0.58 < *r* < -0.55, *P* < 0.11, *n* = 20–23; Exp. 2: -0.69 < *r* < -0.53, *P* < 0.0001, *n* = 20) whilst the correlation was not significantly different from zero in *An. coluzzii* (Exp. 1: -0.37 < *r* < -0.36, *P* < 0.12, *n* = 18; Exp. 2: -0.16 < *r* < 0.29, *P* < 0.085, *n* = 38). These results suggest that upon emergence, in *An. arabiensis* females there is a trade-off between body size and lipid content, whereas in *An. coluzzii* lipid content is relatively constant across body size.

#### Experiment 1

**Fig. 2:**
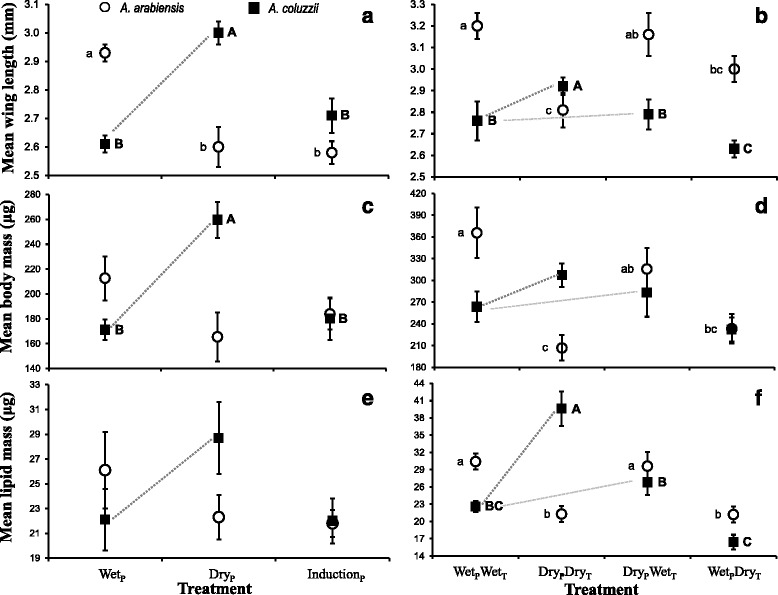
Phenotypic responses of female *A. arabiensis* (white circles) and *A. coluzzii* (black squares) under three photoperiod treatments (Experiment 1; panels **a**, **c**, **e**) and four photoperiod-temperature treatment combinations (Experiment 2; panels **b**, **d**, **f**), described in Fig. 1. Phenotypes examined were wing length (**a**, **b**), body mass (**c**, **d**), and lipid mass (**e**, **f**). Mean ± SE are given for each trait and significantly different values within each species are designated with letters (REGWQ groupings). The grey dotted line denotes the critical comparison between wet-season and dry-season *A. coluzzii* in each experiment

Photoperiod significantly affected female body size (wing length) for both *An. arabiensis* (*F*_(__2,22)_ = 14.3, *P* = 0.0001) and *An. coluzzii* (*F*_(__2,17)_ = 14.3, *P* = 0.0002). However, the direction of the change differed between the species; under the dry-season photoperiod, female *An. arabiensis* were smallest while *An. coluzzii* were largest (Fig. 2a). Female *An. arabiensis* were not significantly different in dry mass at emergence between the three photoperiod treatments (*F*_(__2,22)_ = 2.8, *P* = 0.085), although the trend followed that exhibited by their body size (Fig. 2c). In contrast, female *An. coluzzii* under the dry-season photoperiod had a significantly higher dry mass at emergence than those in either the wet-season or the wet-to-dry transition treatments (*F*_(__2,20)_ = 13.5, *P* = 0.0002; Fig. 2c). The absolute lipid mass at emergence was not significantly different among treatments for both species (*F* < 2.38, *P* > 0.12; Fig. 2e), although the trends are qualitatively similar to those of dry mass. Moreover, relative lipid content was not significantly different between treatments of both species (*F*_(__2,20)_ = 3.45, *P* > 0.059 and *F*_(__2,19)_ = 0.64, *P* > 0.53 for *An. arabiensis* and *An. coluzzii*, respectively; Additional file 1: Figure S2).

#### Experiment 2

Temperature (*F*_(1,19)_ = 13.2, *P* = 0.0023) but not photoperiod alone (*F*_(1,19)_ = 2.3, *P* = 0.15) or the interaction between photoperiod and temperature (*F*_(1,19)_ = 1.2, *P* = 0.30) significantly affected body size (wing length of female *An. arabiensis*. Female *An. arabiensis* raised under the wet-wet treatment were the largest while those raised under the dry-dry treatment were the smallest (Fig. 2a). In contrast, photoperiod alone (*F*_(1,34)_ = 6.8, *P* < 0.014) and the interaction between photoperiod and temperature (*F*_(1,34)_ = 4.4, *P* < 0.04) significantly affected body size (wing length) for female *An. coluzzii*, while temperature alone was non-significant (*F*_(1,34)_ = 0.0, *P* = 0.96). Similar to the results of Exp. 1, female *An. coluzzii* raised under the dry-dry treatment were larger than those raised under the wet-wet treatment (Fig. 2b). Temperature (*F*_(1,19)_ = 20.9, *P* = 0.0003) but not photoperiod (*F*_(1,19)_ = 2.1, *P* = 0.17) or their interaction (*F*_(1,19)_ = 0.2, *P* = 0.66) significantly affected dry mass at emergence for *An. arabiensis* females. Female *An. arabiensis* raised under the wet-season temperature treatments were significantly heavier than those females raised under the dry-season temperature treatments (Fig. 2d). For female *An. coluzzii*, the effect of photoperiod on dry mass at emergence was marginally significant (*F*_(1,34)_ = 4.1, *P* = 0.052), while temperature and the interaction term were both non-significant (*F*_(1,34)_ < 1.4, *P* > 0.24). However, the qualitative differences in dry mass at emergence for *An. coluzzii* were similar to those of Exp. 1 (Fig. 2d). Unlike Exp. 1, body lipid mass at emergence was significantly affected by the treatment for both species (Fig. 2f). For female *An. arabiensis*, lipid mass at emergence was significantly affected by temperature (*F*_(1,19)_ = 25.3, *P* = 0.0001) but not by photoperiod or the interaction term (*F*_(1,19)_ < 0.08, *P* > 0.8); female *An. arabiensis* had higher amounts of lipids under both wet-season temperature treatments as compared with the two dry-season temperature treatments (Fig. 2f). By contrast, for female *An. coluzzii*, photoperiod (*F*_(1,34)_ = 47.4, *P* < 0.0001) and the interaction term (*F*_(1,34)_ = 22.9, *P* < 0.0001), but not temperature alone (*F*_(1,34)_ = 2.7, *P* = 0.11), significantly affected lipid mass at emergence. Female *An. coluzzii* raised under the dry-dry treatment had the highest amount of lipid at emergence (Fig. 2f), similar to the trend seen in Exp. 1 (Fig. 2e). Moreover, relative lipid content was also not significantly different between treatments in *An. arabiensis* (*F*_(1,19)_ < 1.7, *P* > 0.19) but significantly differed between treatments in *An. coluzzii* (*F*_(1,34)_ = 10.1, *P* < 0.0001). Notably, under shorter photoperiod and lower nightly temperature relative lipid content was highest in *An. coluzzii* (Additional file 1: Figure S2).

### Adult longevity

Longevity (from adult ecolosion to death) of a total of 1473 females consisting of 676 and 797 of *An. arabiensis* and *An. coluzzii*, respectively (including 74 and 81 censored mosquitoes, respectively) was measured in Exp. 1. Longevity of a total of 2041 females consisting of 392 and 1657 of *An. arabiensis* and *An. coluzzii*, respectively (with additional 84 and 229 censored mosquitoes, respectively) was measured in Exp. 2. Overall mean longevity of *An. arabiensis* and *An. coluzzii* in the first and second experiments were 19.4 d (SE = 0.39, max = 55 d) and 20.5 d (SE = 0.38, max = 63 d), 26.5 d (SE = 0.52, max = 59 d), and 25.8 d (SE = 0.26, max = 64 d), respectively.

#### Experiment 1

**Fig. 3:**
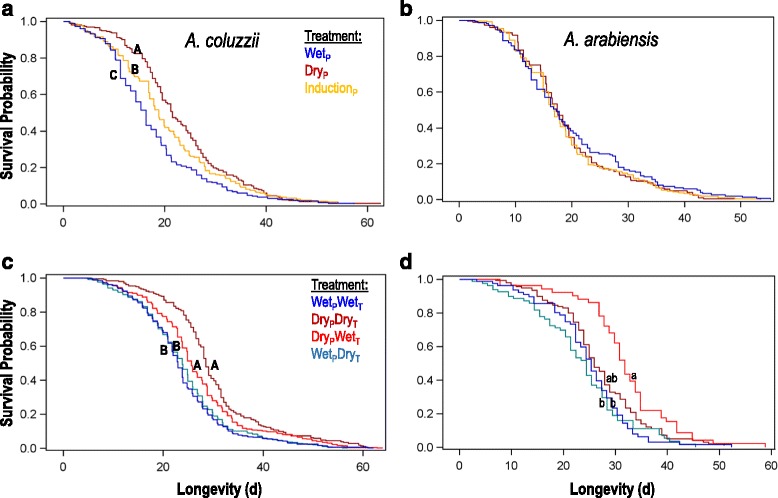
Longevity of female *A. coluzzii* (panels **a**, **c**) and *A. arabiensis* (panels **b**, **d**) under three photoperiod treatments (Experiment 1; panels **a**, **b**) and four photoperiod-temperature treatment combinations (Experiment 2; panels **c**, **d**), described in Fig. 1. Letters designate significantly different values within each species and treatment combination (log-rank test with multiple comparisons)

For *An. arabiensis*, no significant difference in female longevity was found between the three photoperiod treatments tested (Wilcoxon *χ* ^2^ = 0.94, *df* = 2, *P* = 0.59; Fig. 3b), with mean longevity under the wet-season photoperiod at 20.0 d (SE = 0.84), under the transition photoperiod at 19.2 d (SE = 0.62), and under the dry-season photoperiod at 19.3 d (SE = 0.62). In contrast, photoperiod significantly affected longevity of female *An. coluzzii* (Wilcoxon *χ* ^2^ = 30.04, *df* = 2, *P* < 0.0001; Fig. 3a), with dry-season mosquitoes having the highest mean longevity (23.1 d, SE = 0.64), wet-season mosquitoes having the shortest mean longevity (18.0 d, SE = 0.60), and transition photoperiod mosquitoes having intermediate longevity (20.6 d, SE = 0.74).

#### Experiment 2

Unlike Exp. 1, mean longevity of female *An. arabiensis* was significantly affected by treatment (Wilcoxon *χ* ^2^ = 30.2, *df* = 3, *P* < 0.0001), with higher longevity under dry-season photoperiod/wet-season temperature (32.0 d, SE = 1.3), as compared with the wet-season photoperiod/dry season temperature (23.5 d, SE = 1.1), and wet-wet (24.8 d, SE = 0.96) treatments (Fig. 3d). Notably, the dry-dry treatment (27.0 d, SE = 0.77) was intermediate and statistically similar to all other treatments (after Tukey-Kramer multiple test adjustment: *P* > 0.087). For *An. coluzzii*, treatment also significantly affected female longevity (Wilcoxon *χ* ^2^ = 85.7, *df* = 3, *P* < 0.0001; Fig. 3c). In contrast to *An. arabiensis*, for *An. coluzzii* the highest mean longevity was exhibited under the dry/dry treatment (30.1 d, SE = 0.68), which was significantly greater than all other treatments (after Tukey-Kramer multiple test adjustment: *P* < 0.039). The next highest mean longevity was achieved under dry photoperiod/wet temperature (26.9 d, SE = 0.51), which was higher than the two wet-season photoperiod treatments, which were also not significantly different from each other (wet-dry = 24.2 d, SE = 0.46 and wet-wet = 23.8 d, SE = 0.47). Initial adult density did not affect longevity of *An. arabiensis* (*P* > 0.90) or *An. coluzzii* (Wilcoxon *χ* ^2^ = 0.5, *df* = 1, *P* > 0.48).

## Discussion

The mechanisms by which anopheline mosquitoes survive the long dry season in the African Sahel is one of the longest standing questions in medical entomology [58]. Recent studies have indicated that *An. coluzzii* likely aestivates locally while *An. gambiae* and *An. arabiensis* recolonize yearly towards the beginning of the rainy season [8, 20, 25, 27–30, 32, 33]. Additional research continues to gather evidence testing new aspects of this hypothesis, yet, the environmental conditions which initiate aestivation and migration are poorly known for tropical mosquitoes and insect species [6, 21, 34, 59]. The present study aimed to induce aestivation in *An. coluzzii* under laboratory conditions by manipulating photoperiod and temperature. The rationale for this approach is the importance of these environmental factors as cues for adult mosquitoes undergoing diapause [43, 60–69] and the observation that tropical insects from as low latitudes as 9° use photoperiod as a cue to initiative aestivation [45]. Our experimental approach also included a comparison of the response to photoperiod and temperature between the aestivating species *An. coluzzii* and its non-aestivating sibling species, *An. arabiensis*. In the first experiment, we simulated three field-relevant photoperiods: wet-season, dry-season, and wet-dry transition (all under constant temperature of 27 °C). In the second experiment, we combined wet-season and dry-season photoperiods with temperature modulations to mimic natural field conditions during these seasons. These studies were designed to test the hypothesis that short photoperiod and lower nightly temperatures could trigger physiological changes which increase lifespan of the presumably aestivating species *An. coluzzii*, but will not produce such responses in *An. arabiensis* (Table 1). The second experiment not only expanded the factors tested to include nightly lower temperatures which was found to vary sharply during the transition from the wet to the dry season [21], but also allowed assessment of the response’s robustness through its repeatability. The effect of the treatments on longevity arguably provides the best measure of the response of *An. coluzzii* relevant to aestivation.

Our key findings demonstrated that short photoperiod, and to a lesser extent, lower nightly temperature, significantly increased longevity of *An. coluzzii* in both experiments (Fig. 3). The increase in longevity of *An. coluzzii* from wet to dry season photoperiod was modest (30.0 and 24.4%, respectively) but statistically significant (*P* < 0.001). Additionally, body size (wing length) increased in response to short photoperiod in both experiments (Fig. 2a, b), as was previously found in field studies [6, 20, 27, 29, 30, 32] and in accord with a previous laboratory study [34]. The larger adult size was attained despite a shorter larval developmental time (trend in both experiments, but significantly so only in Exp. 1; Fig. 1). Body mass of *An. coluzzii* followed body size (trend in both experiments, but significantly only in Exp. 1). Total lipid mass at emergence was elevated under dry season conditions, but significantly so only in Exp. 2 (Fig. 3). Triacylglyceride lipids are a common form of energy storage during diapause in insects, likely due the relatively low hydration state and high metabolic water yield of these molecules [62]. Thus, reduced pre-adult development time (Fig. 1a), increased body size (Fig. 2a, b), increased body mass (Fig. 2c) and increased lipid mass (Fig. 2f), as well as increased adult longevity (Fig. 3a, c) in response to changes between wet-season and dry-season photoperiod/temperature conditions are all consistent with a coordinated aestivation “syndrome”. These coordinated changes also suggest trade-offs elsewhere in the life-cycle under dry season conditions, which are likely manifest as reduced reproductive output, consistent with the lack of dry season reproduction in nature [21, 32]. These results suggest that *An. coluzzii* has entered a diapause initiation phase in our experiments, at least partly. Nonetheless, these environmental changes alone failed to manifest a *bona fide* aestivating adult recognized by a longevity greater than 3 months, raising the question of what (if any) additional conditions must be met if aestivation is to be manifested?

Under the same conditions, *An. arabiensis* exhibited no consistent increase of longevity (Fig. 3). Specifically, longevity of *An. arabiensis* under dry season conditions was not significantly longer than any other treatment in both experiments (Fig. 2). Moreover, its body size as well as body mass decreased (Fig. 2), in contrast to the response of *An. coluzzii*. The differing responses in this experiment of *An. coluzzii* and *An. arabiensis* to photoperiodic conditions characteristic of wet and dry seasons in the Sahel further supports our hypothesis of alternative life-history responses to dry seasons in nature. The prediction that *An. arabiensis* uses photoperiod to initiate long-distance migration may be more directly tested using assays of flight aptitude rather than longevity. However, the species-specific responses demonstrated in this study underscore the capacity of tropical anophelines to detect subtle environmental cues on the one hand and the role these seasonal changes play as token stimuli for *An. coluzzii* in induction of aestivation, on the other. In Exp. 2, lower nightly temperature did not increased the response of either species in terms of larval developmental time, body size, and mass for *An. coluzzii*. In *An. arabiensis*, lower nightly temperatures yielded no difference in longevity with respect to all other treatments, indicating no response to temperature. However, under short photoperiod, lower nightly temperature led to an increase in mean longevity for *An. coluzzii*. The effect of lower nightly temperatures was not detected under long photoperiod (Fig. 3), suggesting that this effect was not mediated by the lower nightly temperature alone. Additional regimes of temperature and photoperiod might extend longevity further.

In the temperate zone, photoperiod changes provide the primary cue used by insects and mosquitoes in particular to induce diapause and temperature may also play a role, but typically of lesser importance [40, 43, 45, 46]. In mosquitoes, including *Culiseta inornata*, the only species for which a laboratory model of aestivation is available, photoperiod change was sufficient to induce aestivation [63–65, 68, 69]. Furthermore, previous laboratory studies have suggested that short photoperiod results in longer longevity and large body size in (presumably) non-diapausing *An. quadrimaculatus* and *An. crucians* mosquitoes that originated from north Florida [70–72]; yet, the authors could not offer an explanation or an adaptive benefit for these responses. Because the range of both these species includes expansive temperate regions where overwintering diapause must occurs, it is difficult to rule out that the observed responses were indeed linked to diapause. Further, even under mild winter conditions, diapause may be selected for, as is the case of *Drosophila melanogaster* [73]. Additional studies are needed to clarify whether short photoperiod induce extended longevity and larger body size in non-diapausing mosquitoes, however, given the extensive body of evidence connecting these responses to diapause, it seems prudent to connect responses to photoperiod with diapause (aestivation). Accordingly, the extended longevity and larger body size at emergence despite a shorter larval developmental time of *An. coluzzii* in response to 1 h changes in photoperiod are difficult to explain without reference to aestivation.

The seemingly effortless success of early studies on laboratory simulation of aestivation of presumably *An. coluzzii* in Burkina Faso [18] and *An. arabiensis* in Sudan [6] has met with serious challenges for replication. Sadly, the previous authors have provided insufficient details about their experimental procedures. Whether their success in maintaining females of these species between three and 6 months during the dry season was dependent on the particular population, time of the year, or a “secret ingredient” of their procedure, have remained elusive as this and similar investigations have shown [59, 74]. It is possible that more extreme photoperiod and temperature conditions integrated with changes in RH are necessary to produce the diapause initiation phase in full. Additionally, the conditions required for diapause maintenance remain less known. Potentially these include, total darkness, lower temperature, dietary changes, and even changes in gas composition if the shelters used by the species are deep underground. For example, if aestivating mosquitoes shelter in deep termite mounds, they might be exposed not only to mild temperatures and total darkness, but also to unique volatiles. Could such termite nests be under the insectaries of Holstein in Burkina Faso [18] and Omar and Cloudsley-Thompson in the Sudan [6]?

## Conclusions

We compared the responses of *An. coluzzii* and *An. arabiensis* to changes in photoperiod and temperature and specifically aimed to induce aestivation in *An. coluzzii* under shorter photophase and lower nightly temperature, typical of the early dry season. Under dry season conditions, longevity, body size, and total lipids of *An. coluzzii* increased despite a shorter larval developmental time, consistent with a coordinated aestivation “syndrome”. Under the same conditions, longevity of *An. arabiensis* did not increase and its body size decreased. These results provide evidence for a differential response to photoperiod in these species and that *An. coluzzii* has entered a diapause initiation phase in our experiments, at least partly. Nonetheless, these environmental changes alone failed to manifest a *bona fide* aestivating adult recognized by a longevity greater than 3 months, raising the question of what (if any) additional conditions must be met if aestivation is to be manifested?

## Additional files

Additional file 1: Figure S1. Cage design of Experiment 2, with plastic “shelter” in one corner of the cage. **Figure S2.** The relative lipid content of female *An. arabiensis* (white circles) and *An. coluzzii* (black squares) under three photoperiod treatments (Experiment 1; panel **a**) and four photoperiod-temperature treatment combinations (Experiment 2; panel **b**). Least square means ± 95% CI are given for each trait and significantly different values within each species are designated with letters. The grey dotted line denotes the critical comparison between wet-season and dry-season *An. coluzzii* in each experiment. (ZIP 3439 kb)

## Abbreviations

ANOVA: Analysis of variance
CI: Confidence interval
d: Day
L:D: Light:dark
LMVR: Laboratory of Malaria and Vector Research (NIH)
RH: Relative humidity
SE: Standard deviation of the mean
