# Supplementary figures and images for: Photoperiodic responses of Sahelian malaria mosquitoes Anopheles coluzzii and An. arabiensis
Source: Parasit Vectors. 2017 Dec 27;10:621. doi: 10.1186/s13071-017-2556-z (PMC5745990; doi:10.1186/s13071-017-2556-z)

## Slide 1
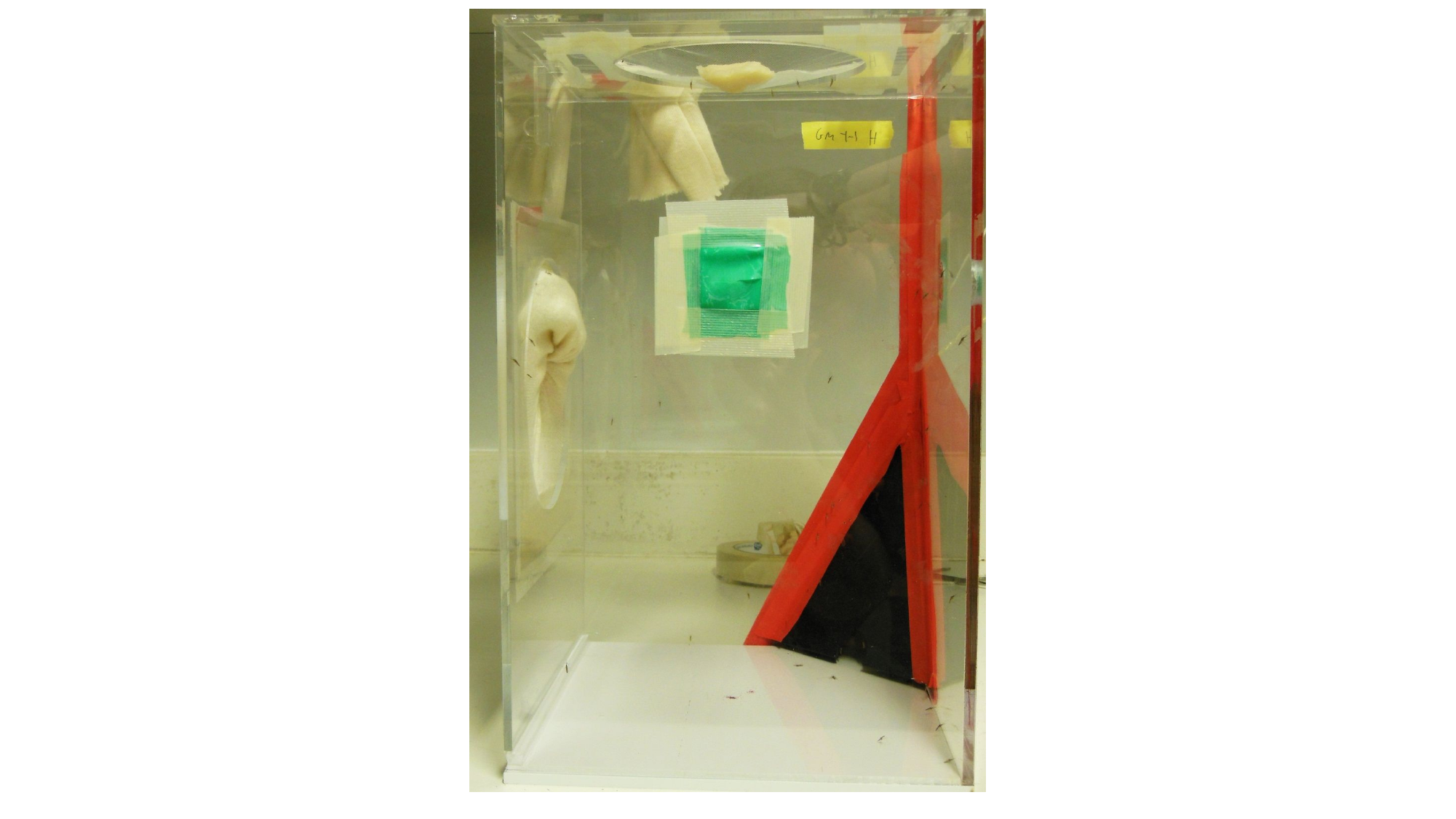

Supplement: Supplementary file 1 — Cage design of Experiment 2, with plastic “shelter” in one corner of the cage. Figure S2. The relative lipid content of female An. arabiensis (white circles) and An. coluzzii (black squares) under three photoperiod treatments (Experiment 1; panel a) and four photoperiod-temperature treatment combinations (Experiment 2; panel b). Least square means ± 95% CI are given for each trait and significantly different values within each species are designated with letters. The grey dotted line denotes the critical comparison between wet-season and dry-season An. coluzzii in each experiment. (ZIP 3439 kb) [file 13071_2017_2556_MOESM1_ESM.zip › HuestisParVectFigS1.pptx]

## Slide 1
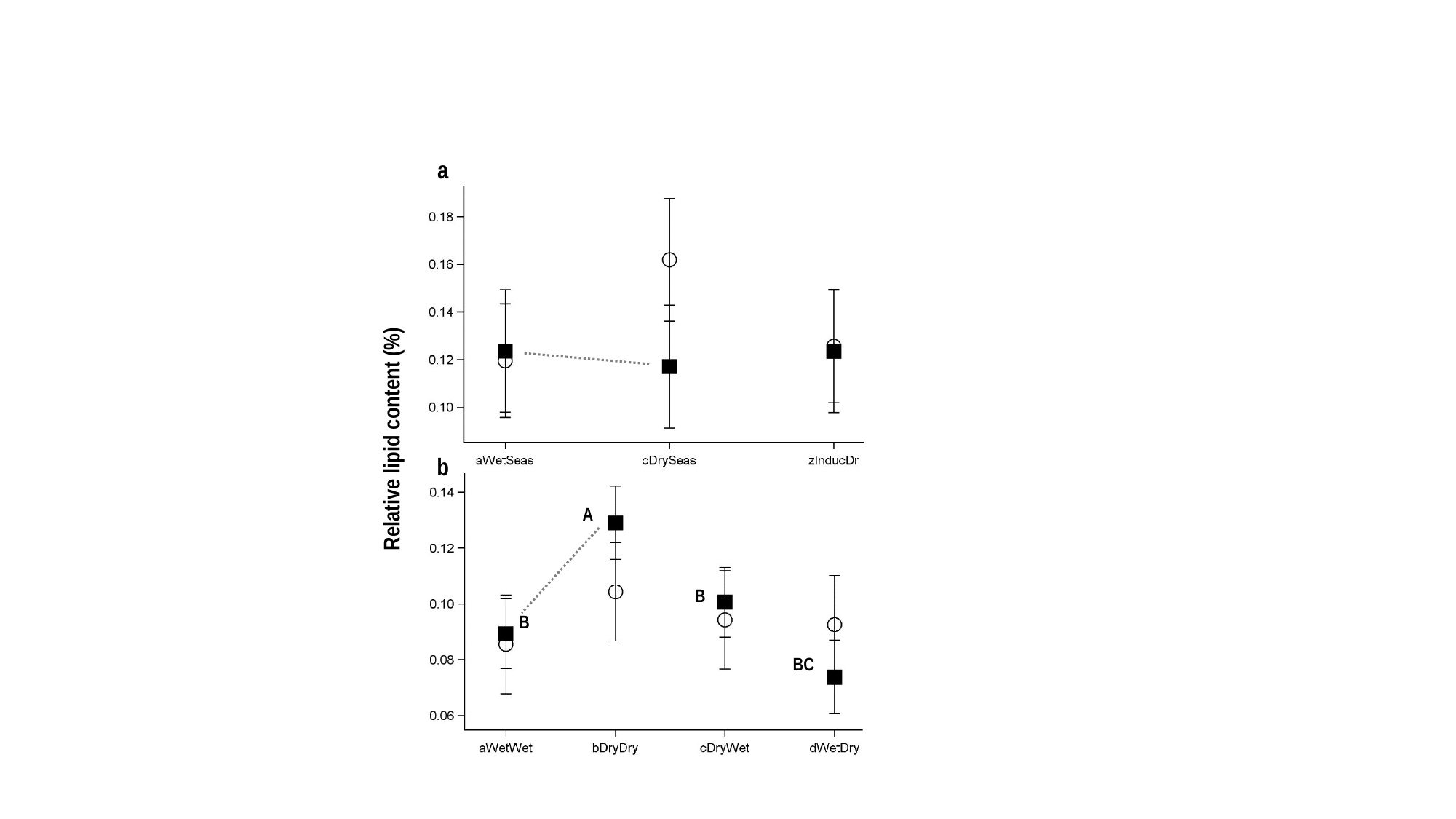

a
Relative lipid content (%)
b
A
B
B
BC

Supplement: Supplementary file 1 — Cage design of Experiment 2, with plastic “shelter” in one corner of the cage. Figure S2. The relative lipid content of female An. arabiensis (white circles) and An. coluzzii (black squares) under three photoperiod treatments (Experiment 1; panel a) and four photoperiod-temperature treatment combinations (Experiment 2; panel b). Least square means ± 95% CI are given for each trait and significantly different values within each species are designated with letters. The grey dotted line denotes the critical comparison between wet-season and dry-season An. coluzzii in each experiment. (ZIP 3439 kb) [file 13071_2017_2556_MOESM1_ESM.zip › HuestisParVectFigS2.pptx]
